# Use of eribulin as an earlier-line chemotherapy for patients with HER2-negative metastatic breast cancer

**DOI:** 10.7150/jca.37670

**Published:** 2020-04-07

**Authors:** Sumito Shingaki, Takahiro Kogawa, Mototsugu Shimokawa, Kenichi Harano, Yoichi Naito, Shota Kusuhara, Yumi Fujimoto, Nobuaki Matsubara, Ako Hosono, Hirofumi Mukai, Tatsuya Onishi, Takashi Hojo, Toru Mukohara

**Affiliations:** 1 Departments of Breast and Medical Oncology, National Cancer Center Hospital East, Kashiwa, Japan; 2 Department of Developmental Therapeutics, National Cancer Center Hospital East, Kashiwa, Japan; 3 Department of Breast Surgery, National Cancer Center Hospital East, Kashiwa, Japan; 4 Cancer Biostatistics Laboratory, Clinical Research Institute, National Kyushu Cancer Center, Fukuoka, Japan; 5 Department of Biostatistics, Yamaguchi University Graduate School of Medicine, Ube, Japan

**Keywords:** Eribulin, Metastatic breast cancer, Chemotherapy, First line chemotherapy

## Abstract

**Background**: Previous prospective studies have shown that eribulin improves the survival in patients with metastatic breast cancer (MBC). However, the optimal timing of its administration to achieve the longest extended survival and the efficacy of using eribulin monotherapy as earlier-line chemotherapy are yet unclear.

**Methods**: We identified all consecutive female patients with MBC who received any chemotherapeutic intervention for metastatic disease at our institution between July 2012 and December 2017, excluding patients with HER2-positive disease. Those who received eribulin monotherapy for MBC were classified under the eribulin cohort, whereas those who never received eribulin were included in the non-eribulin (Non-E) cohort. Among the patients in the eribulin cohort, those who received eribulin as the first- or second-line chemotherapy for MBC were further classified under the earlier-line eribulin (EE), and otherwise classified under the later-line eribulin (LE) cohorts. The survival of patients was assessed using the log-rank test. A multivariable Cox proportional hazards model was used to assess the independent efficacy and timing of eribulin monotherapy. The inverse probability of treatment weighting (IPTW) estimate was utilized to compare the EE and LE cohorts.

**Results**: Of the 507 patients who were initially screened, 226 were included after an intensive chart review: 93, 49, and 84 patients were included in the Non-E, EE, and LE cohorts, respectively. The eribulin cohort showed significantly longer overall survival than the Non-E cohort (30.3 vs. 22.2 months, *p* = 0.0217). No significant difference was observed in the progression-free survival of the EE and LE cohorts (3.4 vs. 4.4 months, *p* = 0.1337) after adjusting for clinically relevant factors using IPTW estimates. LE cohort showed good overall survival (OS) compared with patient group of Non-E and EE by log-rank testing (*p* = 0.0398), although multivariate analysis did not demonstrate eribulin administration timing as an independent prognostic factor of OS. OS was defined from the initiation of first-line chemotherapy date.

**Conclusions**: Our data provided additional insights regarding the use of eribulin monotherapy as earlier-line chemotherapy. However, the optimal timing of eribulin monotherapy for MBC was not determined in the current study.

## Introduction

Survival in patients with metastatic breast cancer (MBC) has improved over the recent decades. Anthracycline- or taxane-based regimens extend the overall survival (OS) in patients with breast cancer; thus, such regimens are used for metastatic disease 1. However, the long-term survival of patients with MBC remains poor, which necessitates effective therapy to improve their quality of life and prolong survival 2.

Eribulin is a non-taxane inhibitor of microtubule dynamics belonging to the halichondrin class of anti-neoplastic agents 3. In addition to its primary anti-neoplastic function, preclinical studies have suggested other potential benefits of these drugs, such as preventing epithelial-mesenchymal transition (EMT) to inhibit cancer stem cell traits and improving the tumor microenvironment by vascular remodeling to reduce drug resistance and metastasis 4-6. The Study 305/EMBRACE has revealed that eribulin as third- or later-line chemotherapy for MBC improved OS compared with the treatment selected by physicians 7. Accordingly, eribulin has been usually administered as third- or later-line chemotherapy for MBC in clinical practice.

Recent phase II single-arm trials have indicated the efficacy and safety of eribulin monotherapy as earlier-line chemotherapy for MBC 8, 9. Furthermore, real-world data obtained by Mougalian et al. have indicated the clinical benefit of eribulin monotherapy as earlier-line treatment for patients with metastatic triple-negative breast cancer (TNBC) 10. However, whether eribulin monotherapy for MBC is more beneficial as earlier-line chemotherapy than as later-line chemotherapy is yet unknown.

Therefore, this study aimed to determine whether eribulin monotherapy as earlier-line chemotherapy, rather than later-line chemotherapy, improves OS in patients with MBC. Furthermore, we assessed whether eribulin improved OS in patients with MBC, including those who received eribulin as first-line chemotherapy.

## Methods

### Patients

**Figure 1:**
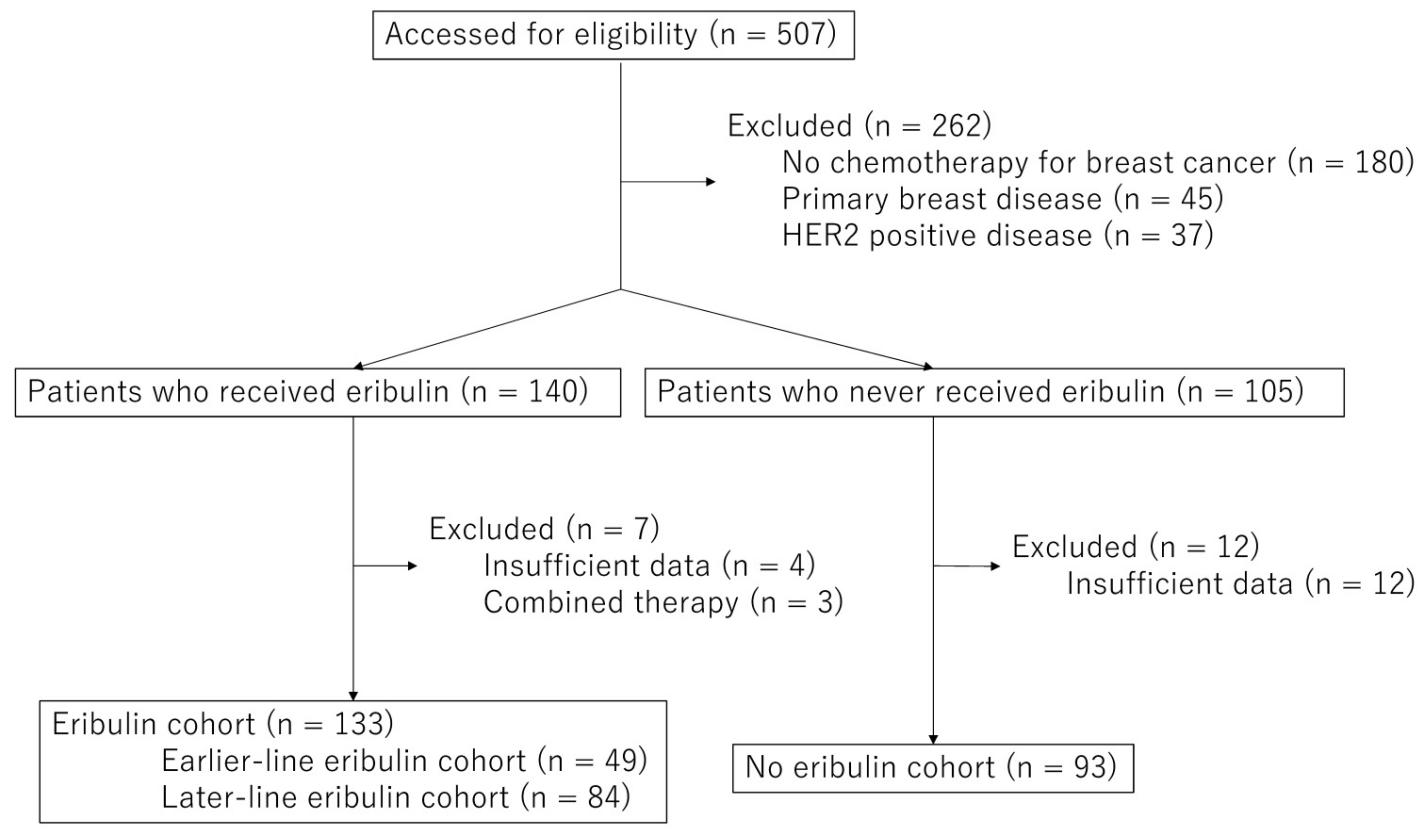
Flow diagram of screening patients in this study. After screening 507 patients, 226 were included for further analyses.

In this single institution retrospective chart review study, we identified all consecutive female patients with MBC (i.e., unresectable local disease and/or distant metastatic disease) who received any chemotherapeutic treatment for metastatic disease and/or unresectable locally advanced disease at the National Cancer Center Hospital East between July 2012 and December 2017 by reviewing the prescription record for eribulin and other chemotherapeutic regimens electronically and by reviewing the departmental database regarding MBC. Metastatic disease or locally advanced disease was confirmed through diagnostic radiography with computed tomography and/or whole body bone scintigraphy. Data were obtained on January 1, 2018, which is the reference date. Patients without MBC and those who never received cytotoxic chemotherapy for breast cancer were excluded. Moreover, those with HER2-postive disease were excluded, considering the evidence regarding the insignificant benefits of eribulin on the survival of patients in this subgroup 2, 10. Because the analyses regarding survival were the main endpoints in the current study, patients whose first-line chemotherapy initiation dates were unavailable and those who received eribulin but whose eribulin monotherapy initiation dates were unavailable were excluded for further analyses. Of the 507 breast cancer patients, we excluded 180 patients who were not treated with cytotoxic chemotherapy, 45 patients whose metastatic disease revealed to be non-metastatic disease, and 37 patients with HER2 positive disease. Additionally, 16 patients whose medical record was not sufficient and 3 patients treated with combination chemotherapy with eriburin (Figure 1, and supplement). This study was conducted in accordance with the declaration of Helsinki and was approved by the institutional review board of the National Cancer Center (protocol number: 2017-431).

### Definitions

The lines of chemotherapies for MBC were considered to be primary metastatic or relapsed breast cancer diagnosis; (neo) adjuvant chemotherapies administered in definitive settings were not counted as one line of chemotherapy for MBC. The eribulin cohort was defined as patients who received eribulin monotherapy for MBC, whereas the non-eribulin (Non-E) cohort was defined as patients who never received eribulin. Among the patients in the eribulin cohort, those who received eribulin as first- or second-line chemotherapy for MBC were classified under the earlier-line eribulin (EE), and otherwise classified under the later-line eribulin (LE) cohorts. Visceral disease was defined as baseline disease during the initiation of the first chemotherapy for advanced disease at any of the following sites: central nervous system, esophagus, liver, lung, peritoneum, pleura, renal, small bowel, stomach, pancreas, colon, rectum, ovary, ascites, pericardial effusion, or spleen 7.

### Statistical analysis

The primary endpoint of this study was OS. OS1 was defined from the initiation of first-line chemotherapy date until any cause of death or censored on the last visit date, and OS2 was defined from the time of initiation of eribulin. And progression-free survival (PFS) was defined from the first administration date of eribulin monotherapy until disease progression or death, and these were estimated using the Kaplan-Meier method. Patients were followed-up until death; those who were alive on the reference date and lost to follow-up were censored during the last available follow-up date. The survival curves of the two groups were compared using the log-rank test. A multivariable Cox proportional hazards model was used to assess the independent efficacy and timing of eribulin monotherapy, thereby adjusting for the following pre-planned fixed characteristics: age during first-line chemotherapy initiation, hormone receptor status, primary metastatic or recurrent disease, and the presence of visceral disease during first-line chemotherapy initiation for MBC. To compare clinical outcomes between the EE and the LE cohorts, we utilized the inverse probability of treatment weighting (IPTW) estimate in which the weights were calculated as the inverse of the propensity score, thereby adjusting for the clinical characteristics that were used for the multivariate analyses. All statistical analyses were conducted using the SAS software version 9.4 (SAS Institute, Cary, NC, USA).

## Results

We included 226 patients after initially screening 507 patients excluding breast cancer patients untreated with chemotherapy (n=180), primary breast disease (n=45), HER2 positive disease (n=37), and 19 patients with insufficient data and those who received eribulin as combined chemotherapies were excluded (Figure 1). In total, 133 patients were classified under the eribulin cohort and 93 under the No-E cohort. Among the patients in the eribulin cohort, 49 (33.9%) and 84 (66.1%) were further classified under the EE and LE cohorts, respectively. The characteristics of the participants during chemotherapy initiation for MBC are depicted in Table 1.

**Figure 2:**
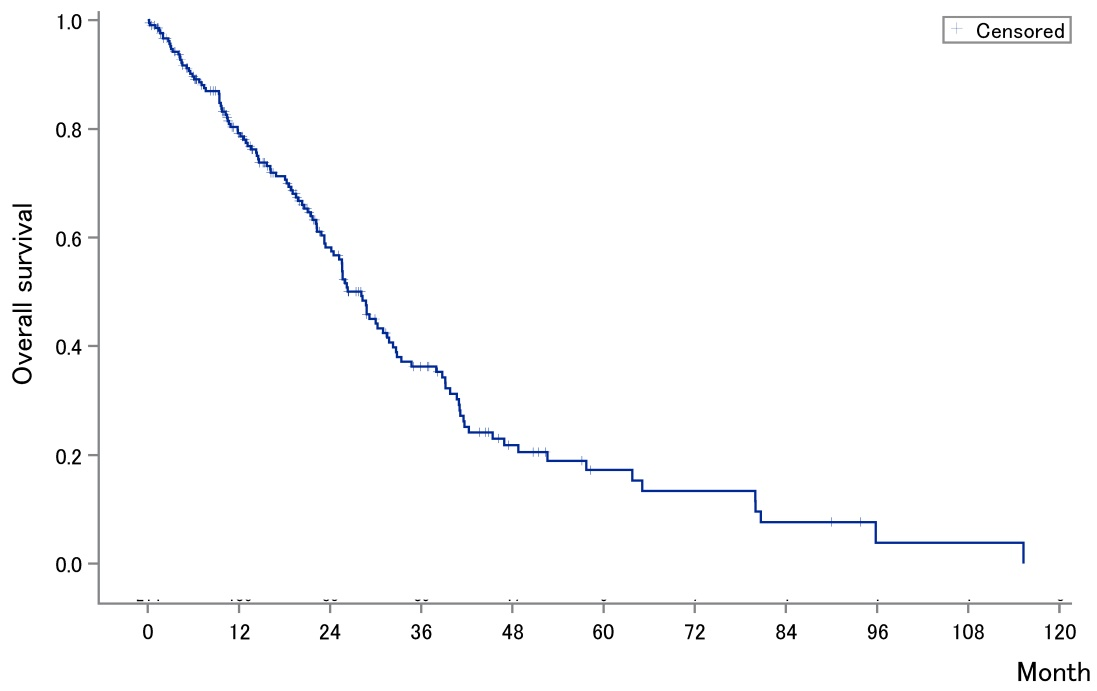
Kaplan-Meier estimates for overall survival (OS) after first-line chemotherapy for metastatic breast cancer. The median OS after first-line chemotherapy for MBC was 27.6 months (95% confidence interval, 3.0-4.3 months).

**Figure 3:**
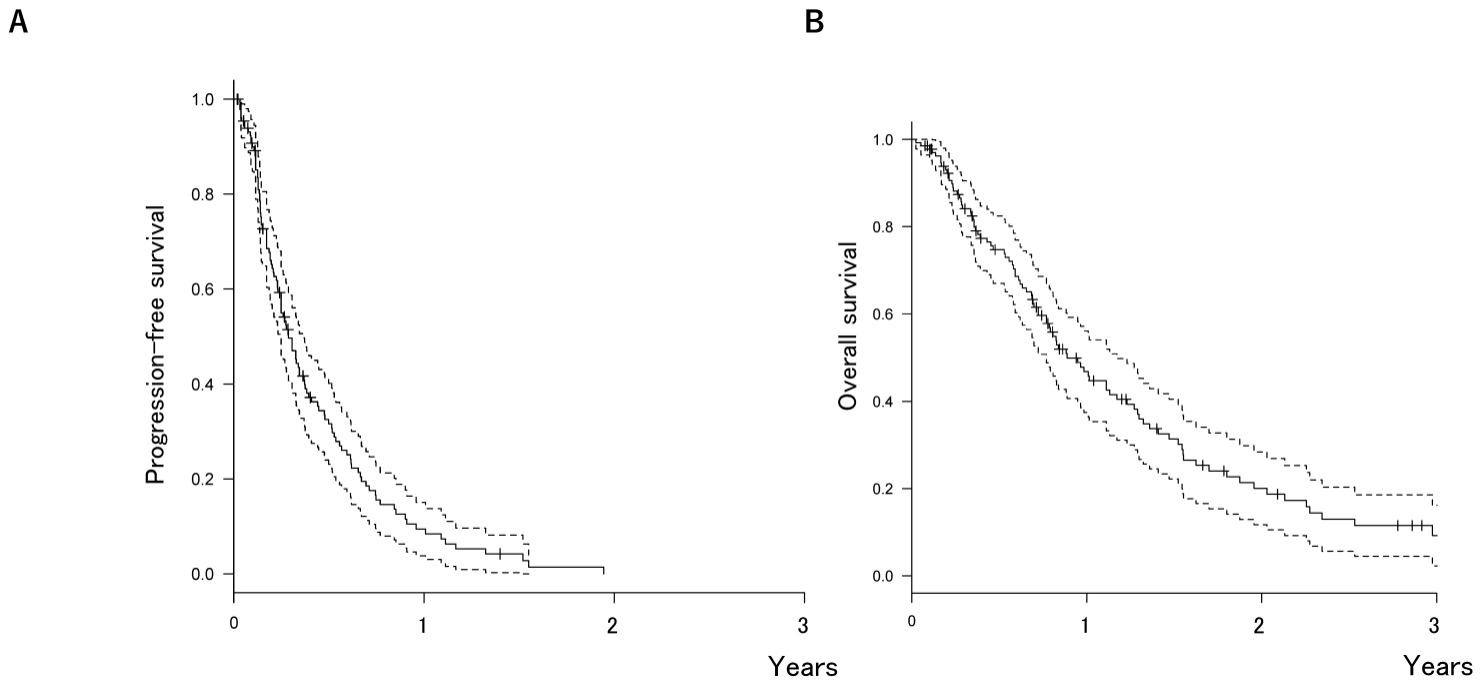
Kaplan-Meier estimates for progression-free survival (PFS) (A) and overall survival (OS) (B) after eribulin monotherapy. The median PFS and OS were 3.5 months (95% confidence interval [CI], 3.0-4.3 months) and 10.7 months (95% CI, 9.2-14.1 months), respectively.

**Figure 4:**
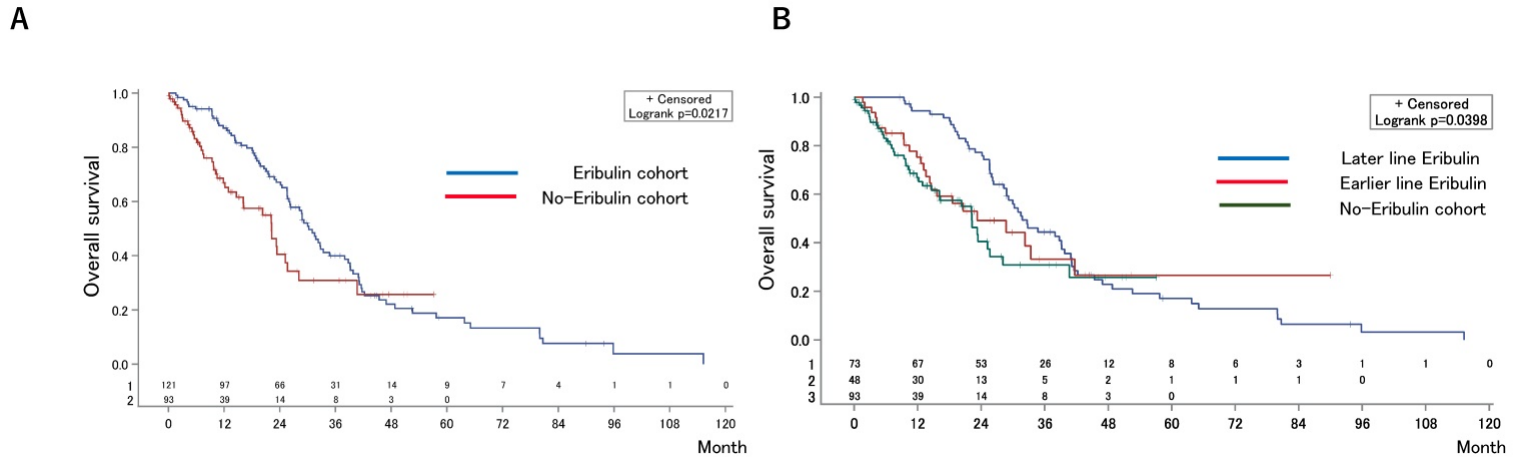
Kaplan-Meier estimates for overall survival (OS) after the first-line chemotherapy in the eribulin and non-eribulin cohorts (A) and in the earlier-eribulin, later-eribulin, and non-eribulin cohorts (B). The median OS values were 30.3 months for the eribulin cohort and 22.2 months for the non-eribulin cohort. Furthermore, those for the earlier- and later-eribulin cohorts were 23.3 and 31.8 months, respectively.

**Figure 5:**
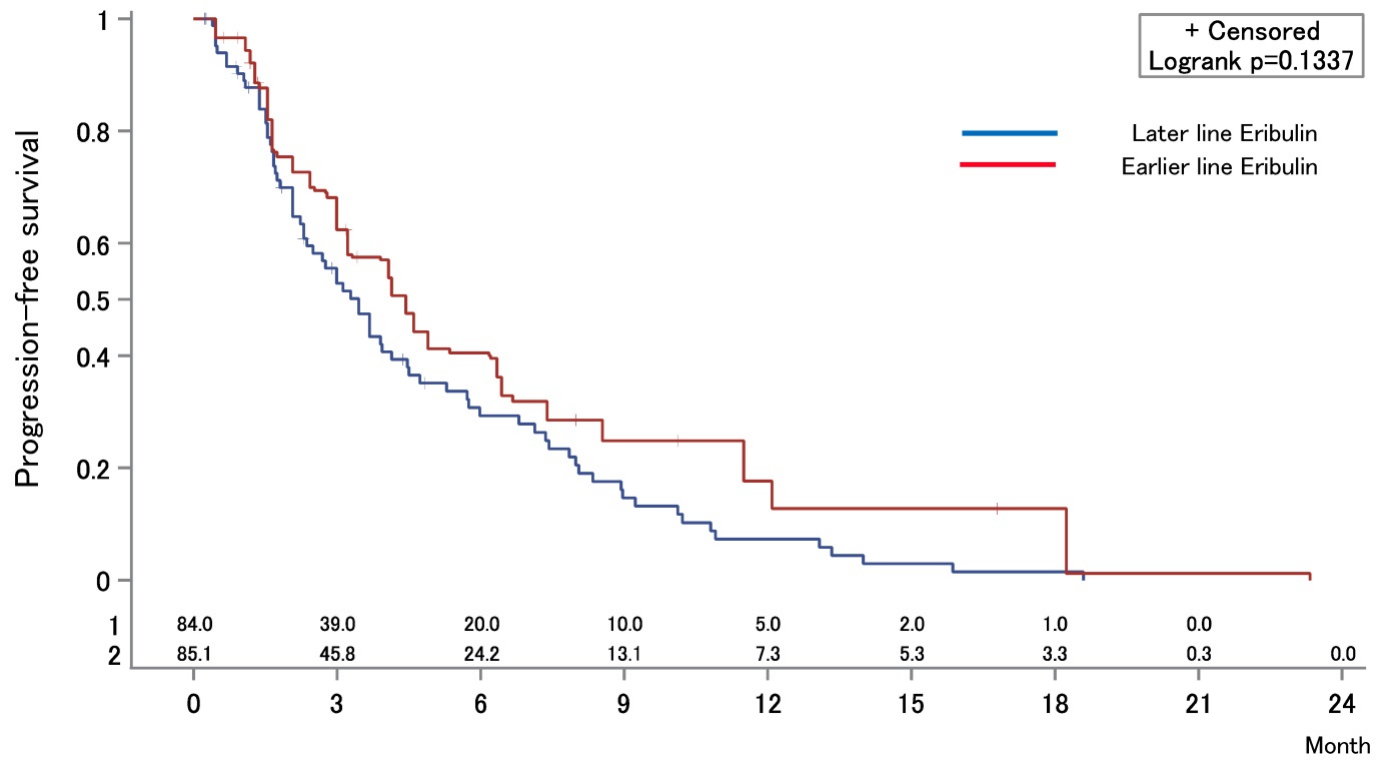
Inverse probability of treatment weighting estimate for progression-free survival (PFS) after eribulin monotherapy. The median PFS values were 3.4 months in the earlier-eribulin cohort and 4.4 months in the later-eribulin cohorts (log-rank test, *p* = 0.1337).

The median OS after first-line chemotherapy (OS1) for MBC was 28.1 months (95% confidence interval [CI], 24.2-31.5 months) (Figures 2). The median PFS and OS after eribulin monotherapy initiation (OS2) in the eribulin cohort were 3.5 months (95% CI, 3.0-4.3 months) and 10.7 months (95% CI, 9.2-14.1 months), respectively (Figure 3). The eribulin cohort showed significantly longer OS1 than the No-E cohort (30.3 vs. 22.2 months, *p* = 0.0217; Figure 4A). The OS1 values were 31.8, 23.3, and 22.2 months in the LE, EE, and Non-E cohorts, respectively (*p* = 0.0398; Figure 4B). The multivariate analysis revealed that earlier-line eribulin treatment did not significantly contribute to OS1 (Table 2). No significant difference was observed in terms of PFS in the LE and EE cohorts (3.4 vs. 4.4 months, *p* = 0.1337) after adjusting for clinically relevant factors using IPTW estimates (Figure 5).

## Discussion

We showed that the eribulin cohort had a longer OS1 than the non-E cohort, which is consistent with the results of a phase III trial 7, and longer survival was achieved regardless of eribulin administration timing for HER2-negative disease. And, no significant difference was observed in the survival outcomes of the EE and LE cohorts after adjusting for prognostic factors using multivariate analysis and IPTW estimates. The median survival endpoints following eribulin monotherapy in the current cohort (PFS, 3.5 months; OS2, 10.7 months) were comparable to those of previous randomized phase III trials (PFS, 3.7 months; OS, 13.1 months) 7. In contrast, the optimal timing of eribulin monotherapy is yet unclear. Notably, heavily pretreated patients in the LE cohort conspicuously achieved comparable PFS to that of the EE cohort, considering the general principle in cancer therapy: cancer gets more refractory when it has received more treatment. This finding is consistent with the report showing that prior chemotherapies were not associated with the efficacy of eribulin 11. In addition, the survival benefit of eribulin monotherapy has been reported only in patients who received prior chemotherapy at least once in prospective studies 12. Collectively, eribulin should be selected as second- or later-line chemotherapy in daily clinical practice.

In contrast, there is accumulating evidence regarding the use of eribulin as earlier-line chemotherapy. A clinical study has indicated that patients with TNBC who received eribulin as earlier-line chemotherapy had longer PFS 12; this result is consistent with preclinical findings showing that epithelial-mesenchymal transition, an aggressive mechanism in TNBC, is alleviated by eribulin 4. Although our current study included only 24 patients with TNBC and did not produce enough statistical power, our approach must be used when comparing the EE and LE cohorts after adjusting for prognostic factors using multivariate analysis and IPTW estimates in a larger cohort of patients with TNBC.

The limitations of the current study include its retrospective nature in data collection, which may result in the potential deviation of the disease characteristics in the cohorts. First, patients in the LE cohort were more likely to have indolent disease such that they survive for a sufficiently long duration to receive more than two lines of chemotherapies, whereas patients in the EE cohort were more likely to have aggressive disease such that they were unable to receive more than two lines of chemotherapies. And we could not fully include whether patient had anthracycline and/or taxane if they had primary breast cancer before. These could present a bias toward the EE cohort. To minimize the potential bias, we have performed survival analyses by comparing the two groups after adjusting for the prognostic factors using the IPTW estimation. Another limitation may be the small sample size, resulting in insufficient statistical power for detecting clinical significance.

In conclusion, our data provided additional insights regarding the use of eribulin monotherapy as earlier-line chemotherapy. However, the optimal timing of eribulin monotherapy for MBC was not determined in the current study.

## Supplementary Material

Supplementary information.

## Figures and Tables

**Table 1:** Patient characteristics at the initiationof chemotherapy

Characteristic		No eribulin		Eribulin (n = 133)			Earlier-line vs. Later-line	No eribulin vs. Eribulin
		(n = 93)	Event	Earlier-line (n = 49)	Event	Later-line (n =84)	*p*-value	*p*-value
Age, years		62 (35-85)	57 (31-77)					*p* = 0.0518
			38	57.5 (33-75)	74	57 (31-77)	*p* = 0.7935	
Hormone receptor	Positive	69 (74%)	109 (82%)					*p* = 0.1604
			23	32 (65%)	67	77 (92%)	*p* = 0.0001	
	Negative	24 (26%)	24 (18%)					
			15	17 (35%)	7	7 (8%)		
Disease characteristic	Primary metastatic disease	38 (41%)	30 (23 %)					*p* = 0.0032
			5	8 (16%)	20	22 (26%)	*p* = 0.1892	
	Relapsed disease	55 (59%)	103 (77%)					
			33	41 (84%)	54	62 (74%)		
Site of disease	Visceral disease	82 (88%)	104 (78%)					*p* = 0.0531
			30	39 (80%)	57	65 (77%)	*p* = 0.7658	
	Non-visceral disease only	11 (12%)	29 (22%)					
			8	10 (20%)	17	19 (23%)		

**Table 2:** A multivariate analysis identifying significant factors for overall survival 1

Variable	Univariate				Multivariate		
	HR	95% CI	*p*-value		HR	95% CI	*p*-value
Age	1.004	0.988-1.021	0.6203		1.002	0.986-1.018	0.7949
Hormone receptor status (positive vs. negative)	0.557	0.365-0.849	0.0065		0.588	0.374-0.924	0.0212
Disease characteristic (primary metastatic disease vs. relapsed disease )	1.195	0.817-1.749	0.3584		1.088	0.739-1.601	0.6699
Site of disease (visceral disease vs. non-visceral disease only)	2.117	1.247-3.596	0.0055		2.216	1.296-3.787	0.0036
Eribulin-line (later-line vs. no eribulin)	0.537	0.331-0.870	0.0116		0.664	0.398-1.107	0.1166
Eribulin-line (earlier-line vs no eribulin)	0.711	0.463-1.089	0.1171		0.808	0.521-1.254	0.3422

CI, confidence interval; HR, hazard ratio.
