# Supplementary material for: Use of eribulin as an earlier-line chemotherapy for patients with HER2-negative metastatic breast cancer
Source: J Cancer. 2020 Apr 7;11(14):4099–105. doi: 10.7150/jca.37670 (PMC7196254; doi:10.7150/jca.37670)
Supplement: Supplementary file 1 — Supplementary information. [file jcav11p4099s1.pdf]

Supplemental

Inclusion criteria

1. Patients were female breast cancer patients with unresectable locally advanced disease and/or distant metastatic disease.
2. Metastatic disease both recurrent disease and de novo stage IV
3. Histlogically proven breast cancer
4. Human epidermal receptor 2 (HER2) negative disease
5. Patient who was treated with chemotherapy at the timing of patient inclusion reference date.

Excluding criteria

1. Patients whose initial chemotherapy administration date was not clear.
2. Patients whose eribulin administration date was not clear if they were treated with eribulin at any timing.
3. Patient characteristics (i.e. age, hormone receptor status, recurrent/de novo stage IV, visceral/non-visceral disease status) was insufficient.
